# Engineering a CRISPR-Mediated Dual Signal Amplification-Based Biosensor for miRNA Determination

**DOI:** 10.3390/bios16010017

**Published:** 2025-12-24

**Authors:** Zhixian Liang, Jie Zhang, Shaohui Zhang

**Affiliations:** 1 National Engineering Research Center for Healthcare Devices & Guangdong Provincial Key Laboratory of Medical Electronic Instruments and Materials, Institute of Biological and Medical Engineering, Guangdong Academy of Sciences, Guangzhou 510316, China; zhangjie_gdas@foxmail.com (J.Z.); shzhang2016@sinano.ac.cn (S.Z.); 2 School of Pharmaceutical Science, Sun Yat-sen University, Guangzhou 510006, China

**Keywords:** miRNA-21, CRISPR, self-priming hairpin isothermal amplification, TdT mediated polymerization labeling

## Abstract

MicroRNAs, pivotal regulators of gene expression and physiology, serve as reliable biomarkers for early cancer diagnosis and therapy. As one of the earliest discovered miRNAs in the human genome, miRNA-21 provides critical information for early cancer diagnosis, drug therapy, and prognosis. In this work, we harness CRISPR as a bridge to integrate target-induced self-priming hairpin isothermal amplification (SIAM) with terminal transferase (TdT) polymerization labeling, constructing a facile, straightforward electrochemical biosensor for sensitive miRNA-21 detection. Unlike conventional single-strand template-based exponential amplification (EXPAR), the SIAM hairpin undergoes target triggered intramolecular conformational change, initiating extension and strand displacement reactions that suppress nonspecific dimer formation and lower background current. Notably, the assay requires only a single probe, enabling unidirectional signal amplification while nonspecific reactions caused by system complexity. The generated SIAM products activate the Cas12a/crRNA complex to trans-cleave PO_4_^3−^ modified single-stranded DNAs (ssDNAs); the resulting 3′ hydroxyl ssDNAs are subsequently labeled by TdT, with the assistance of SA-HRP catalyzing hydrogen peroxide, achieving robust signal amplification. Under optimized conditions, the cathodic current exhibits a logarithmic relationship with miRNA concentrations from 20 fM to 5.0 × 10^8^ fM, with a detection limit of 9.2 fM. The biosensor successfully quantified miRNA-21 in commercial serum samples and biological lysates, demonstrating its potential for cancer diagnostics and therapy.

## 1. Introduction

As one of the earliest identified miRNAs in the human genome, miRNA-21 (miR-21) is highly expressed in a variety of tumors, such as breast, lung, and pancreatic cancers [1]. Within cells, miR-21 targets p53, transforming growth factor-β, and several key components of the mitochondrial apoptosis-tumor-suppressor network. Studies have demonstrated that upregulation of miR-21 in cancer cells effectively blocks p53-mediated apoptosis, thereby shielding the cells from DNA-damage-induced death caused by chemotherapeutic agents [2]. Consequently, miR-21 is widely regarded as an oncogene closely linked to cancer cell proliferation, differentiation, apoptosis, invasion, and metastasis. Developing accurate and sensitive detection methods for miR-21 is therefore crucial for cancer diagnosis and therapy.

Traditional miRNA detection methods, such as Northern blotting [3], reverse transcription-PCR (RT-PCR) [4], and microarrays [5], are widely used but suffer from complex primer design, high cost, and low efficiency, largely because miRNAs are short, highly similar in sequence, and present at low abundance [6,7]. To improve analytical performance, signal amplification strategies such as exponential amplification reaction [8] (EXPAR), rolling circle amplification (RCA), catalytic hairpin assembly (CHA) [9], and hybridization chain reaction (HCR) [10] have garnered significant attention. Among these, EXPAR is extensively utilized due to its exponential amplification capability. In contrast to single-strand-template-based EXPAR, self-priming hairpin isothermal amplification (SIAM) utilizes hairpin probes as substrates. By leveraging intramolecular conformational changes to drive the extension process, this approach effectively minimizes nonspecific binding caused by random collisions of single strands, thereby significantly enhancing biosensor sensitivity [11]. Consequently, SIAM offers isothermal operation, rapidity, high efficiency, strong sensitivity, and operational simplicity, making it a valuable tool in biomedical research and clinical diagnostics [12]. Nevertheless, after thermal termination, SIAM products would re-associate with the excess substrate, limiting signal output.

To overcome this challenge, clustered regularly interspaced short palindromic repeats (CRISPR) system, capable of recognizing both single- and double-stranded oligonucleotides, has emerged as a novel amplification strategy for biosensor construction [13]. The system comprises Cas proteins and CRISPR RNA (crRNA), which work synergistically to achieve precise and efficient target recognition. Owing to their high sensitivity and broad applicability, CRISPR/Cas-based detection methods have attracted considerable attention [14,15,16]. In particular, the CRISPR–Cas12a platform employs crRNA to bind target DNA and subsequently activates Cas12a, which indiscriminately cleaves any single-stranded DNA (ssDNA) present in the reaction mixture [17]. Exploiting this collateral cleavage activity, several nucleotide-detection platforms, such as SPARC [18], ID-CRISPR [19], HOLMES [20,21], and SHERLOCK [22,23], have been developed. The exceptionally rapid enzymatic turnover of Cas12a confers outstanding sensitivity and specificity, enabling exponential signal amplification in molecular diagnostics. As the mechanistic understanding of Cas12a proteases deepens, the potential of CRISPR–Cas12a systems in the field of biosensing has been demonstrated in practical disease diagnosis scenarios [24]. Consequently, leveraging CRISPR-mediated signal amplification may pave the way for novel, portable, and rapid sensors for early detection of cancer.

Herein, we propose an electrochemical sensor that leverages CRISPR-mediated SIAM and terminal transferase (TdT) polymerization labeling for the quantitative detection of the cancer biomarker miR-21. To suppress nonspecific SIAM reactions and enhance sensitivity, we concealed the stem base of the SIAM reaction substrate, which undergoes conformational change upon target binding, within the stem of the original hairpin. The synergistic action of the endonuclease and Klenow fragment (KF) shortens the response time and markedly enhances reaction efficiency. Moreover, CRISPR technology bridges the SIAM reaction with the TdT-mediated polymerization labeling reaction, endowing the sensor with high specificity and excellent sensitivity. Additionally, The TdT step enables the Cas12a-cleaved probe to generate multiple biotin-modified single-stranded DNAs, achieving multiplex signal amplification together with SA-HRP. The facile, fast-responding analytical assay showed high sensitivity and stability, as well as low detection limits, by exploiting the synergistic effects of CRISPR-mediated SIAM and TdT polymerization labeling.

## 2. Materials and Methods

### 2.1. Materials and Reagent

Tris (2-carboxyethy) phosphine hydrochloride (TCEP), 6-mercaptohexanol (MCH), bovine serum albumin (BSA), Tris(hydroxymethyl)aminomethane (Tris), and hydroquinone (HQ) were obtained from Aladdin Chemistry Co., Ltd. (Shanghai, China). The microRNA extraction kit, one-step RT-qPCR Kit, agarose B, dNTP (10 mM), diethylpyrocarbonate-treated water (DEPC-DW), 4S Gelred, 1 × TE buffer (10 mM Tris-HCl, 1.0 mM EDTA, pH 8.0), phosphate buffer saline (PBS), and 0.4 mg/mL SA-HRP were purchased from Sangon Biotech Co., Ltd. (Shanghai, China). Nuclease-free water, Nb.BbvCI (Nb, 10 U/μL), Klenow Fragment (3′→5′ exo-, KF, 5 U/μL), EnGen Lba Cas12a (Cpf1) nuclease (100 μM), terminal deoxynucleotidyl transferase (TdT, 20 U/μL), NEBuffer 1, 10 × TdT buffer, and CoCl_2_ solution (2.5 mM) were provided by New England Biolabs Inc. (Beijing, China); 2 × SYBR green mix, RNase Inhibitor (40 U/μL), and Annealing buffer (AB) were acquired from Beyotime Biotech Co., Ltd. (Shanghai, China). Human breast cancer cells (MCF-7), biotin-11-dUTP (10 mM) was ordered from Thermo Fisher Scientific Co., Ltd. (Shanghai, China). Human normal lung epithelial cell line (BEAS-2B), Human lung cancer cells (A549) was purchased from Fenghui Biotechnology Co., Ltd. (Hunan, China). All oligonucleotides (Table S1) used in this study were synthesized by Invitrogen Biotech. Co., Ltd. (Shanghai, China). The lyophilized powder of DNA was diluted with 1 × TE buffer, and the lyophilized powder of RNA was diluted with DEPC-DW.

### 2.2. Apparatus and Measurements

All electrochemical measurements were conducted on a CHI 660E electrochemical workstation (CH Instrument, Shanghai, China). A conventional three-electrode system was employed for all electrochemical experiments. The working electrode was a bare or modified gold disk electrode with a diameter of 3 mm. An Ag/AgCl electrode (3 M KCl) and a platinum wire served as the reference and counter electrodes, respectively (Gaoss Union, Wuhan, China). The concentration of oligonucleotides was quantified using a Nanodrop spectrophotometer (Thermo Scientific, Waltham, MA, USA).The agarose gel visualization was viewed by Tanon Multi Series Fluorescence and Chemiluminescence Imaging System (Tanon, Shanghai, China).

### 2.3. The Preparation of SIAM and CRISPR/Cas12 System

The SIAM reaction substrate was prepared by diluting oligonucleotide HP to 1 µM in 1× AB, followed by annealing on a PCR thermocycler (95 °C for 5 min, then cooling to 25 °C). The resulting solution was stored at 4 °C until use.

Preparation of SIAM reaction: the SIAM reaction was performed in a 40 μL solution by mixing 27.3 μL of DEPC-DW, 2 μL of dNTP (10 mM), 1.5 μL of the pretreated HP solution (1 μM), 2 μL of 10 × NEBuffer 2, 2 μL of 10 × rCutSmart Buffer, 0.4 μL of KF (5 U/μL), 0.7 μL of Nb (10 U/μL), and 4 μL of the target analyte solution. The mixture was incubated at 37 °C for 40 min, then chilled on ice for later use.

Preparation of CRISPR/Cas12a system: Before the reaction, a 20 μL pre-mix solution was prepared in advance. This pre-mix solution was obtained by mixing 9.5 μL of nuclease-free water, 2 μL NEBuffer r2.1, 1 μL RNase Inhibitor (40 U/μL), 2.5 μL Lba Cas12a (1 μM), and 3 μL crRNA (1 μM) and placed at 37 °C for 10 min. Subsequently, 20 μL of the reaction solution (containing 10 μL of SIAM reaction product, 2 μL of NEBuffer r2.1, and 8 μL of nuclease-free water) was added to the above 20 μL mixture to obtain the CRISPR-mediated SIAM system.

### 2.4. Electrode Modification

Prior to use, gold electrodes (GEs) were immersed in a freshly prepared piranha solution (30% H_2_O_2_: 98% H_2_SO_4_ = 1:3, *v*/*v*) for 20 min and rinsed with deionized water. After conventional polishing and cleaning, the electrodes were activated in 0.5 M H_2_SO_4_ to proceed with electrochemical scanning between −0.2 and 1.5 V and nitrogen-dried. Subsequently, 8 μL of SP solution (1 mM TCEP treated, 1 μM) was dropped on the pretreated GE surface and incubated overnight to establish a well-ordered interface. After the modified GE was blocked by 1 mM MCH and 2% BSA to prevent nonspecific adsorption of DNA and enzymes, the CRISPR-mediated SIAM solution was dispensed onto the modified GE surface and incubated at 37 °C for 40 min to induce trans-cleavage of the SP probe.

After gentle PBS washing, 10 μL of the extension solution comprising 1 μL 10 × TdT buffer, 1 μL CoCl_2_, 1 μL TdT (2.5 U/μL), 1 μL dNTP (1 mM), 1 μL biotin-11-dUTP (100 μM), and 5 μL deionized water was cast onto the surface of the prepared electrode and incubated at 37 °C for 80 min. A brief PBS rinse removed loosely bound biotin-11-dUTP. Finally, 6 µL of 1% BSA containing SA-HRP (10 µg/mL) was deposited on the electrode for 20 min to construct the biosensor. Square-wave voltammetry (SWV) was performed in PBS containing 2 mM H_2_O_2_ and 3 mM HQ, with an amplitude of 25 mV, frequency of 15 Hz, and a potential scan from 0.2 V to −0.5 V. Electrochemical impedance spectroscopy (EIS) was conducted in PBS containing 5 mM Fe(CN)_6_^3−/4−^ with an amplitude of 5 mV, initial potential of 0.277 V, and frequency ranging from 0.1 Hz to 10^5^ Hz.

### 2.5. Cell Culture

Cell lines including MCF-7 and BEAS-2B were cultured in a CO_2_ (5%, *v*/*v*) atmosphere incubator (Thermo Scientific, Waltham, MA, USA) at 37 °C with DMEM culture (Corning, New York, NY, USA) medium containing fetal bovine serum (10%, *v*/*v*), penicillin (1%, *v*/*v*), and streptomycin (1%, *v*/*v*). The A549 cells were cultured in RPMI-1640 (Life Technologies) under the same conditions. Once the cells were confirmed to be in good growth condition, they were digested with trypsin and counted. Total RNAs were then extracted using an RNA extraction kit (Sangon, Shanghai, China).

## 3. Results and Discussions

### 3.1. The Principle of the Biosensor

**Scheme 1:**
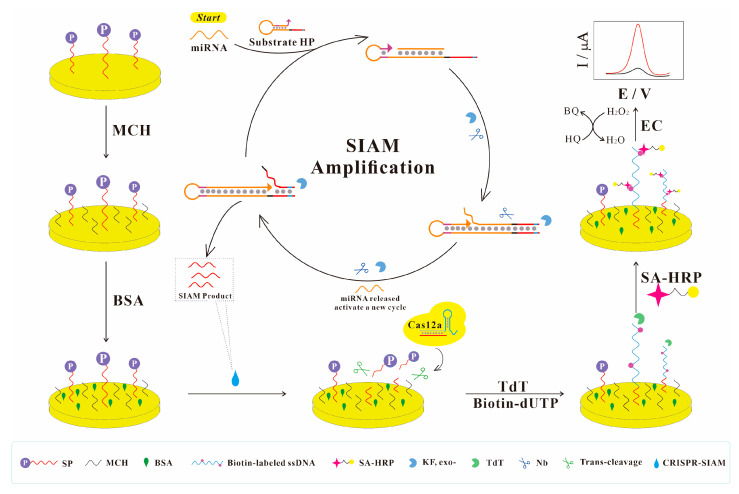
The principle of the constructed biosensor based on CRISPR mediated dual signal amplification.

In this paper, integration of a CRISPR-mediated SIAM reaction with enzyme-driven polymerization labeling was devised for the ultrasensitive detection of cancer-related miRNA. As illustrated in Scheme 1, the biosensor comprises three core components: a substrate probe (SP), the CRISPR-triggered SIAM reaction, and a subsequent polymerization labeling step.

First and foremost, PO_4_^3-^-modified oligonucleotides (SP) were immobilized on the electrode to serve as a platform for CRISPR trans-cleavage and TdT-mediated extension. After blocking residual sites on the electrode surface with MCH and BSA, respectively, the CRISPR-mediated SIAM reaction solution was incubated on the electrode surface. This enables the substrate DNA to undergo trans-cleavage by activated Cas12a, exposing 3′-OH groups at the ends of SP. In the SIAM reaction system, the hairpin (16 nt stem) serving as the reaction substrate hybridizes with the target, promoting the intramolecular conformational change to form a new hairpin (6 nt stem, Figure S1), inducing KF to extend along the 3′-terminus and releasing the target to activate a new SIAM reaction. Concurrently, endonuclease Nb cleaves the specific sequence (CCTCAGC), initiating a strand-displacement reaction. As the cyclic amplification proceeds, abundant ssDNA accumulates, binds crRNA, and further activates Cas12a-mediated trans-cleavage, leading to extensive cleavage of the SP on the electrode surface.

At the end of the SIAM reaction, the single-stranded products that were thermally terminated may recombine with the hairpin substrate. It remains unclear whether the distance between the Protospacer Adjacent Motif (PAM) and the 5′-end influences Cas12a trans-cleavage. To address this, we optimized the hairpin substrate (HP) by adjusting the 5′-PAM spacing, thereby enhancing trans-cleavage efficiency and lowering the sensor’s detection limit. Under TdT and biotin-11-dUTP, the cleaved 3′-OH termini are extended with biotin-labeled ssDNA. Streptavidin-HRP binds to the biotinylated strands and catalyzes the H_2_O_2_-HQ system, finally realizing multiple signal amplification.

### 3.2. Agarose Gel Electrophoresis Analysis of SIAM

Agarose gel analysis was employed for the preliminary characterization of SIAM. As shown in Figure S2, Lane 1 displays the HP band. Upon addition of miR-21, a new band appears, corresponding to the hairpin/miR-21 complex (Lane 2). The subsequent addition of KF and Nb individually leads to extension and cleavage, respectively, generating distinct complexes (Lane 4 and Lane 6). When KF and Nb are present together, the HP substrate is completely consumed, and the HP band disappears (Lane 6). In contrast, no new band is observed in Lane 5, and the HP band remains unchanged in the absence of the target, confirming the feasibility of the SIAM reaction strategy.

### 3.3. Electrochemical Characterization of the Constructed Biosensor

**Figure 1:**
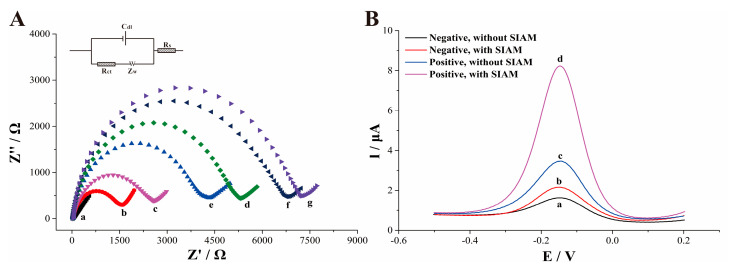
(**A**) Nyquist plots of the developed biosensor. (a) GE, (b) SP/GE, (c) MCH/SP/GE, (d) BSA/MCH/SP/GE, (e) CRISPR-SIAM/BSA/MCH/SP/GE, (f) TdT labeling/CRISPR-SIAM/BSA/MCH/SP/GE, (g) SA-HRP/TdT labeling/CRISPR-SIAM/BSA/MCH/SP/GE. (**B**) SWV response of (a) SA-HRP/TdT labeling/CRISPR/BSA/MCH/SP/GE without target, (b) SA-HRP/TdT labeling/CRISPR-SIAM/BSA/MCH/SP/GE without target, (c) SA-HRP/TdT labeling/CRISPR/BSA/MCH/SP/GE with equimolar concentration of SIAM reaction products, (d) SA-HRP/TdT labeling/CRISPR-SIAM/BSA/MCH/SP/GE with target in the PBS solution (100 mM, pH 7.0) with 3 mM HQ and 2 mM H_2_O_2_. The concentrations of HP, SIAM Product, and target were 37.5 nM, 10 pM, and 10 pM, respectively.

In order to characterize the stepwise assembly process on the surface of the established platform, electrochemical impedance spectroscopy (EIS) was adopted in PBS containing 5 mM Fe(CN)_6_^3−/4−^. In the Nyquist plot, the diameter of the high-frequency semicircle corresponds to the electron transfer resistance (R_et_). As shown in Figure 1, at the outset of the experiment, the bare gold electrode (curve a) exhibits a nearly linear impedance spectrum, indicating superior electron transfer relative to the modified electrodes. Immobilization of PO_4_^3−^-modified oligonucleotides (SP) on the pretreated gold surface via Au-S bonds introduces a negatively charged phosphate backbone, which repels the anionic Fe(CN)_6_^3−^/^4−^ redox probe and forms an insulating layer that increases the charge-transfer resistance (curve b). Since MCH is an electronically inert body and BSA is negatively charged at neutral pH, R_et_ respectively increased to 2500 Ω and 5300 Ω after MCH and BSA blocking (curves c and d). During incubation in the CRISPR-mediated SIAM reaction solution, abundant SIAM products are recognized by the Cas12a/crRNA complex, triggering its trans-cleavage activity that removes the SP probe from the electrode. The loss of phosphate groups diminishes electrostatic repulsion, facilitates electron transfer, and reduces impedance (curve e). Under the action of TdT, the extension of the residual SP single strand endows the electrode surface with stronger negative electrostatic repulsion, expanding the high-frequency semicircle to 6700 Ω (curve f). Finally, immobilization of SA-HRP further elevates the impedance to 7200 Ω (curve g), confirming the complete construction of the biosensor.

Figure 1B illustrates the SWVs of various signal amplification strategies in PBS (100 mM, pH 7.0) bubbled with nitrogen. In the absence of the target, two weak cathodic peaks appear (curves a and b), corresponding to the electrochemical reduction of benzoquinone (BQ) generated by nonspecific adsorption of HRP on the electrode, which catalyzes the H_2_O_2_-HQ redox reaction. When the SIAM reaction is omitted and the biosensor relies solely on TdT-mediated polymerization labeling, the CRISPR–Cas12a system is activated by adding an equimolar concentration of SIAM reaction products. TdT extends the oligonucleotide only at the 3′-OH terminus on the electrode surface, and SA-HRP is subsequently introduced, yielding a peak current of 2.7 µA (curve c). However, when combining CRISPR-mediated SIAM reactions with polymerization labeling, the sensor’s peak current increases to 2.74-fold of the previous value (curve d). This enhancement stems from the synergistic amplification of the CRISPR-mediated SIAM reaction and the enzyme-labeled polymerization. CRISPR serves as a bridge that links the SIAM recycle amplification to the TdT-triggered enzyme labeling. Activated Cas12a removes the phosphate group from the probe’s 3′-terminus on the electrode, exposing 3′-OH groups that become substrates for TdT, which then elongate a biotin-modified sequence, achieving multiple amplification steps. Therefore, the established biosensor may be a proper electrochemical platform for detection of miR-21.

### 3.4. Optimization of the Experiment Conditions

**Figure 2:**
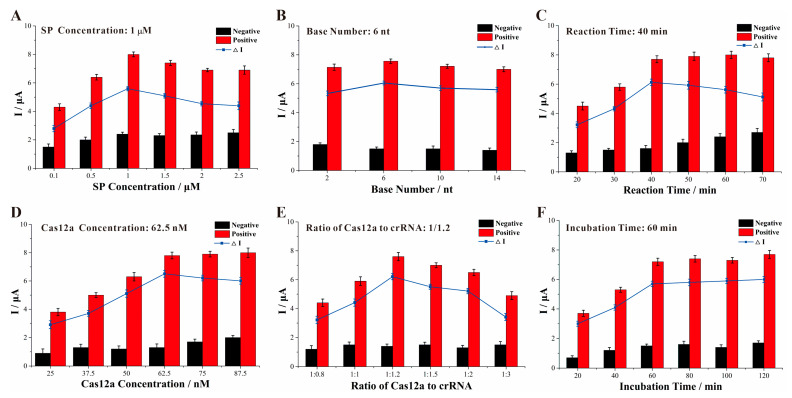
Assessment of the effect of (**A**) SP concentration, (**B**) the base number away from PAM sequence, (**C**) the reaction time of SIAM, (**D**) the concentration of Cas12a, (**E**) the concentration ratio of Cas12a/crRNA, and (**F**) the incubation time of the CRISPR system on SWV signals (n = 3). SWV was conducted in PBS solution containing 3 mM HQ and 2 mM H_2_O_2_ (n = 3, Error Bars: Standard Deviation); the miR-21 concentration was 10 pM.

The biosensor primarily consists of three key components: SP probe, CRISPR-mediated SIAM reaction, and enzyme-polymerization labeling step. The concentration of the SP probe, which serves as the substrate for the subsequent TdT polymerization labeling, critically determines the electrochemical sensor’s performance. To optimize this performance, we employed SWV to the effect of varying SP probe concentrations on sensor performance in PBS solution containing 3 mM HQ and 2 mM H_2_O_2_. As illustrated in Figure 2A, the current difference (ΔI) increases with probe concentration, reaching maximum at 1 µM. Beyond this concentration, further increases in probe concentration lead to a decline in peak currents. This trend likely reflects the balance between the 3′-OH substrate and dUTP in the TdT reaction; excessive 3′-OH primers can hinder efficient incorporation of biotin-11-dUTP by TdT at the probe terminus. Consequently, a 1 µM SP probe concentration was chosen for all subsequent experiments.

The integration of CRISPR-mediated SIAM with an enzyme-catalyzed polymerization labeling reaction significantly enhances the sensitivity of electrochemical sensors. CRISPR–Cas12a is capable of acting on both double-stranded DNA (dsDNA) and single-stranded DNA (ssDNA). When encountering the hairpin-structured SIAM substrate, the Cas12a/crRNA complex initially recognizes the PAM sequence within the dsDNA region. To investigate whether the absence of dsDNA downstream of the PAM sequence influences trans-cleavage activity, we systematically examined the effect of the base count adjacent to the 5′-end of the PAM sequence on the electrochemical performance of CRISPR-mediated SIAM. As illustrated in Figure 2B, nucleotides located 2–14 nt upstream of the PAM sequence can induce trans-cleavage activity, with the maximum current response ΔI observed when the PAM sequence is flanked by 6 nt at the 5′-end. This optimal signal may be attributed to steric hindrance effects, wherein longer 5′-terminal extensions could restrict Cas12a cleavage efficiency [24]. Based on these findings, the HP-6 nt substrate was selected for subsequent experiments. Moreover, SIAM exhibits a cyclic amplification effect. In the absence of a target, nonspecific SIAM reactions are amplified by the CRISPR system and enzymatic polymerization labeling, leading to a high background current. Hence, controlling the SIAM reaction time is essential for regulating the blank signal. Figure 2C shows that ΔI increases with reaction time, reaching a peak at 40 min; thus, 40 min was chosen as the optimal SIAM incubation period.

In the CRISPR system, both the concentration of Cas12a proteins and the protein/crRNA ratio dictate activation efficiency, thereby modulating trans-cleavage activity. We optimized Cas12a concentration and the Cas12a/crRNA complex ratio, assessing their impact on sensor response by recording square wave voltametric signals. Cas protein levels were varied, from 25 nM to 87.5 nM. As shown in Figure 2D, trans-cleavage efficiency increased with protein concentration, accompanied by a larger cathodic current difference. However, concentrations above 62.5 nM triggered nonspecific reactions, reducing the current difference. This may be attributed to the fact that excess Cas12a, while boosting reaction speed, also generates false positives. Consequently, we selected 67.5 nM Cas12a for subsequent 40 µL CRISPR-mediated SIAM assays. The Cas12a-to-crRNA ratio likewise influences target recognition and activation efficiency. We examined various ratios and their effects on the voltametric response. Figure 2E shows that increasing crRNA improves recognition and peak current difference up to an optimal 1:1.2 ratio. Beyond this point, excess free crRNA binds its complement but fails to activate the CRISPR system, diminishing trans-cleavage efficiency. Therefore, a 1:1.2 Cas12a/crRNA ratio was adopted for all further experiments.

The incubation time of the CRISPR-mediated SIAM reaction on the electrode surface directly determines the quantity of the substrate probe SP that is cleaved, thereby influencing the square wave voltametric signal of the sensor. Experiments examining incubation time in the amplified system produced the expected trends (Figure 2F). As incubation time increased, the reduction peak current for positive reactions rose, while that for negative reactions remained essentially unchanged. At 60 min, the current difference reached its maximum, indicating that reaction equilibrium had been attained. Consequently, all subsequent experiments employed a 60-min incubation for the CRISPR-mediated SIAM reaction.

**Figure 3:**
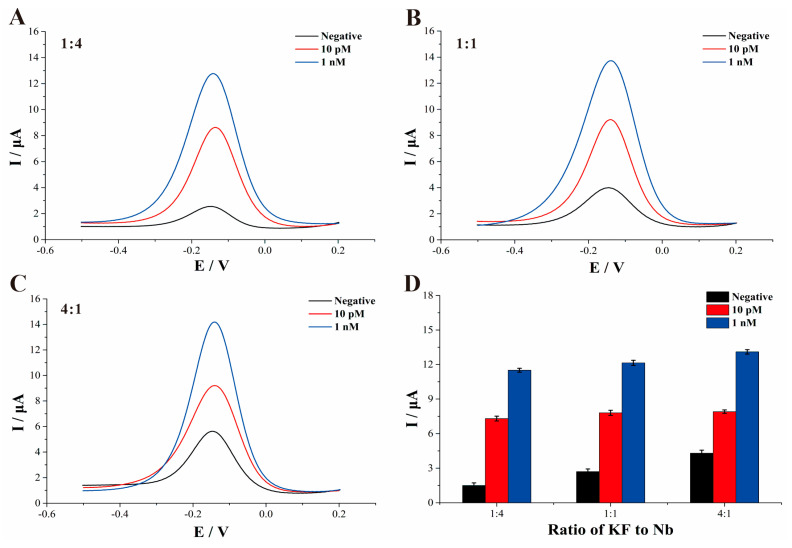
SWV curves for KF:Nb ratios of (**A**) 1:4, (**B**) 1:1, and (**C**) 4:1. (**D**) The influence of different proportions of KF and Nb on the square wave voltammogram signal in SIAM (n = 3, Error Bars: Standard Deviation).

Finally, in our previous study on the effects of varying nucleases Nb (5 U–9 U) and polymerase KF (1–4 U) on real-time fluorescence Ct values in exponential rolling circling amplification (ERCA) reactions [25], we discovered that different enzyme combinations could detect targets with low concentration. Considering reagent consumption, reaction time, and nonspecific background, we selected a mixture of 2 U KF and 7 U Nb for further work. In this work, we first examined the impact of different KF/Nb ratios (1:4, 1:1, 4:1) at varying target concentrations (0, 10 pM, 1 nM) on SWV signals (Figure 3A–C). It was observed that when KF units exceeded Nb, nonspecific reactions increased (Figure 3D). These phenomena may be attributed to the fact that a high concentration of KF will initiate an extension reaction starting from the nonspecific cleavage sites created by Nb, leading to increased negative currents and reduced sensitivity. This finding aligns with other EXPAR-based strategies [26,27,28].

### 3.5. Analysis Performance of miR-21 Detection

**Figure 4:**
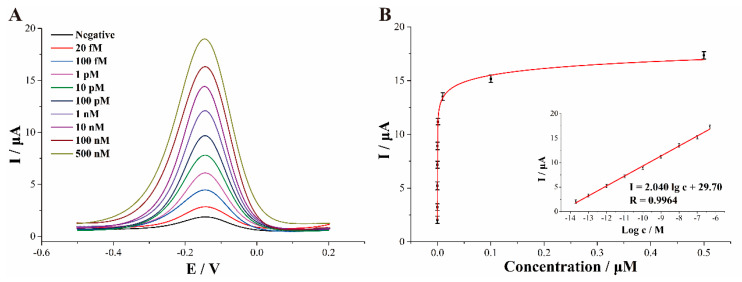
(**A**) SWV curves responding to different miR-21 concentrations. (**B**) Linear relationship between the current variation and the logarithm of the miR-21 concentration.

To assess the detection performance of the fabricated miR-21 sensor, we recorded SWVs under optimized conditions and examined the correlation between the sensor current and miR-21 concentrations. Figure 4 displays the cathodic current response of the sensor to miR-21, ranging from 20 fM to 500 nM. As shown in Figure 4A, the current signal increases proportionally with the target concentration. Figure 4B illustrates the relationship between the reduction current and miR-21 concentration, revealing a linear dependence of the cathodic peak current on the logarithm of concentration with the regression equation I (μA) = 2.040 Lg c (M) + 29.7 and a regression coefficient of 0.9964. Using the 3σ criterion, the limit of detection was calculated to be 9.2 fM. Compared with recent miRNA biosensors (Table 1), our sensor exhibits a wider linear range and a lower detection limit, attributable to (i) the CRISPR/Cas system, which confers high specificity; (ii) optimization of the SIAM reaction to suppress nonspecific interactions; and (iii) TdT-mediated labeling that extends biotin-modified strands on the electrode, enabling streptavidin–HRP catalysis of the H_2_O_2_–HQ system for multiple signal amplification.

### 3.6. Stability, Reproducibility, and Selectivity of the Constructed Biosensor

**Figure 5:**
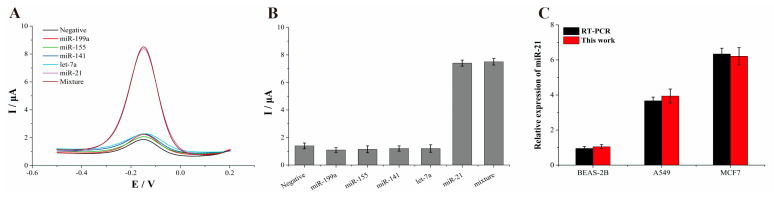
(**A**) SWV of the constructed biosensor with interferers (100 pM) and target (10 pM). (**B**) Selectivity of the biosensor with different sequences. (**C**) Detection of miR-21 from different cell line lysates. n = 3, Error Bars: Standard Deviation.

An ideal biosensor should exhibit outstanding selectivity, reproducibility, and long-term stability. To assess stability, the SP-modified biosensor was immersed in PBS and stored at 4 °C for two weeks. After this period, the electrodes were retrieved and used for subsequent miR-21 detection. The signal intensity retained 87.9% of that obtained with a freshly prepared sensor, with a relative standard deviation (RSD) of 3.3%, confirming the sensor’s stability. Five electrodes were independently fabricated and tested for miR-21 under identical conditions; the RSD across these measurements was 3.9%, indicating excellent reproducibility. Typically, members of the miRNA family share similar sequences with high homology. To visually assess the selectivity of this invention toward miR-21, we performed SWV on interferents at 10-fold concentrations (three miR-21 family members: miR-141, miR-155, miR-199a, and let-7a) (Figure 5A). As anticipated, the current responses of these non-target miRNAs were virtually indistinguishable from the negative control, even at elevated concentrations, demonstrating that they do not interfere with miR-21 detection (Figure 5B). This confirms the sensor’s high selectivity toward its target.

### 3.7. Application in Commercial Simulated Serum Samples and Biological Lysates

To evaluate the applicability and potential clinical value of the miR-21 electrochemical biosensor in complex matrices, spiked experiments were conducted in simulated serum (BZ279, Biochemazone^TM,^, Beijing, China). Serum samples were spiked with miR-21 at various concentrations, diluted 5-fold, and then analyzed with assembled electrochemical sensors to determine the actual miRNA levels. Each sample was tested in triplicate to evaluate any matrix effects on sensor performance. As shown in Table 2, we spiked serum samples with miRNA concentrations of 50 fM, 500 fM, and 5 pM, achieving recoveries of 101.8%, 101.6%, and 104.0%, respectively. The RSD ranged from 2.9% to 5.3%, confirming that complex matrices do not interfere with the miR-21 electrochemical sensor.

Previous studies have demonstrated that miR-21 is highly expressed in patients with lung and ovarian cancers [41,42,43]. To validate the proof-of-concept of this biosensor for real clinical sample analysis, we analyzed human cell line samples derived from diagnosed A549 and MCF-7 cells, along with BEAS-2B cells as controls (Figure 5C). Total RNA was extracted using the SanPrep Column microRNA Extraction Kit after cell counting. As expected, the miR-21 concentration in lung and ovarian cancer cells was significantly higher than that in healthy control cells (*t*-test, *p* < 0.0001). Furthermore, we validated the results of the proposed method using qRT-PCR. The results obtained by the biosensor showed good agreement with the expression levels determined by qRT-PCR for each sample, suggesting that miR-21 can function as a tumor biomarker for the early diagnosis of lung and ovarian cancer.

## 4. Conclusions

This study presents a straightforward and intuitive electrochemical biosensor for miRNA detection, which innovatively integrates CRISPR-mediated SIAM with enzyme-polymerization labeling. The key advancement lies in employing CRISPR technology as a bridge that seamlessly connects the SIAM reaction with the enzymatic polymerization labeling step, thereby enhancing both specificity and signal output. Through systematic optimization of the CRISPR-SIAM system, we significantly improved reaction efficiency and achieved a lower LOD. In parallel, TdT introduces multiple biotin-labeled dUTP sites, providing abundant attachment points for HRP. The immobilized HRP then catalyzes the H_2_O_2_-HQ system, amplifying the electrochemical signal generated from BQ. Under optimal conditions, the cathodic current response shows a linear relationship with the logarithm of miR-21 concentration, achieving a detection limit as low as 9.2 fM. The biosensor exhibits excellent reproducibility, stability, and high selectivity against homologous miRNAs. Compared with current miRNA detection methodologies, our approach offers a wider linear range and a significantly improved detection limit. In summary, this sensor demonstrates high sensitivity and selectivity for miR-21, enabling accurate quantification in simulated serum samples. It thus represents a promising tool for early cancer diagnosis and prognostic assessment.

Although this sensor has made significant progress in miRNA detection, the electrochemical platform itself exhibits poor tolerance to undiluted biological sample matrices, posing considerable challenges for direct clinical application. In the future, the next generation of miRNA electrochemical biosensors can be driven by the convergence of artificial intelligence (AI) and microfluidic technology. Machine-learning algorithms could automatically correct baseline drift, extracting subtle features and predicting optimal assay parameters in real time, thereby enhancing sensitivity and robustness. Meanwhile, microfluidic chips can miniaturize sample handling: on-chip lysis, miRNA extraction, and purification would eliminate matrix effects that currently limit performance in undiluted biological fluids. The synergy of AI-driven signal processing with sophisticated microfluidic workflows promises a new generation of electrochemical biosensors that are faster, more accurate, and readily deployable in clinical settings.

## Figures and Tables

**Table 1:** Comparison of the electrochemical system with other miRNA biosensors that have been reported.

Detection Method	Amplification Method	Target	Linear Range	LOD	Ref.
COL	SIAM and DNAzyme	miR-155	1 pM~10 nM	0.41 pM	[29]
COL	CRISPR–Cas12a and nanozymes	miR-21	1 pM~500 pM	0.5 pM	[30]
COL	Cascade branch migration-triggered strand displacement amplification with G4 structures	miR-155	0.5 pM~2.5 nM	0.28 pM	[31]
SERS	Interfacial reaction system CHA on AuNPs	miR-21	5 fM~1 nM	3.47 fM	[32]
SERS	Entropy-driven circuit and the CRISPR/Cas system	miR-21	100 fM~500 nM	43.8 fM	[33]
FL	DNA hairpins transition triggered strand displacement amplification	miR-21	100 fM~100 nM	58 fM	[34]
FL	Magnetic beads and GO-assisted enzyme-free signal amplification	Let-7a	50 pM~500 nM	15 pM	[35]
ECL	3D DNA walker and RCA	miR-21	1 fM~1 nM	0.14 fM	[36]
ECL	Ru(bpy)_2_(L)^4+^@MOF-5 and CHA	miR-21	10 fM~1 nM	7.2 fM	[37]
PEC	DNA walker, CRISPR–Cas12a, Cu_2_O NCs-enhanced BiVO_4_	miR-21	100 fM~10 nM	35.3 fM	[38]
EC	Exo III-driven recycling and HCR	miRNA-133a	100 fM~1000 pM	26.1 fM	[39]
EC	Methylene blue encapsulated in liposomes	miR-21	200 fM~0.2 μM	85 fM	[40]
EC	CRISPR-mediated SIAM and TdT extension	miR-21	20 fM~500 nM	9.2 fM	This work

COL: Colorimetry, SERS: Surface Enhancement of Raman Scattering, FL: Fluorescence, ECL: Electochemiluminescence, PEC: Photoelectrochemistry, EC: Electrochemistry.

**Table 2:** Detection of miR-21 in simulated serum (n = 3).

Sample	Added/fM	Found/fM			Recovery/%	RSD/%
		1	2	3		
1	50.0	47.9	53.2	51.7	101.8	5.3
2	5.00 × 10^2^	5.12 × 10^2^	5.27 × 10^2^	4.87 × 10^2^	101.6	4.0
3	5.00 × 10^3^	5.07 × 10^3^	5.37 × 10^3^	5.18 × 10^3^	104.0	2.9

## Data Availability

The data presented in this study are available on request from the corresponding author.

## Supplementary Materials

The following supporting information can be downloaded at: https://www.mdpi.com/article/10.3390/bios16010017/s1, Figure S1: Schematic diagram of the conformational change occurring in the secondary structure of the SIAM reaction substrate HP-6nt before reaction and after binding to miR-21; Figure S2: Agarose gel electrophoretic analysis of SIAM reaction; Table S1: Sequence information of the oligonucleotides.
