# Supplementary material for: Engineering a CRISPR-Mediated Dual Signal Amplification-Based Biosensor for miRNA Determination
Source: Biosensors (Basel). 2025 Dec 24;16(1):17. doi: 10.3390/bios16010017 (PMC12838993; doi:10.3390/bios16010017)
Supplement: Supplementary file 1 [file biosensors-16-00017-s001.zip › biosensors-4018848-supplementary.pdf]

biosensors

Supplementary Materials

Engineering a CRISPR-Mediated Dual Signal Amplification-Based Biosensor for miRNA Determination

Zhixian Liang [1,2,*], Jie Zhang [1] and Shaohui Zhang [1]

1 National Engineering Research Center for Healthcare Devices & Guangdong Provincial Key Laboratory of Medical Electronic Instruments and Materials, Institute of Biological and Medical Engineering, Guangdong Academy of Sciences, Guangzhou 510316, China; zhangjie_gdas@foxmail.com (J.Z.); shzhang2016@sinano.ac.cn (S.Z.)
2 School of Pharmaceutical Science, Sun Yat-sen University, Guangzhou 510006, China
* Correspondence: liangzhx8@alumni.sysu.edu.cn

Figure

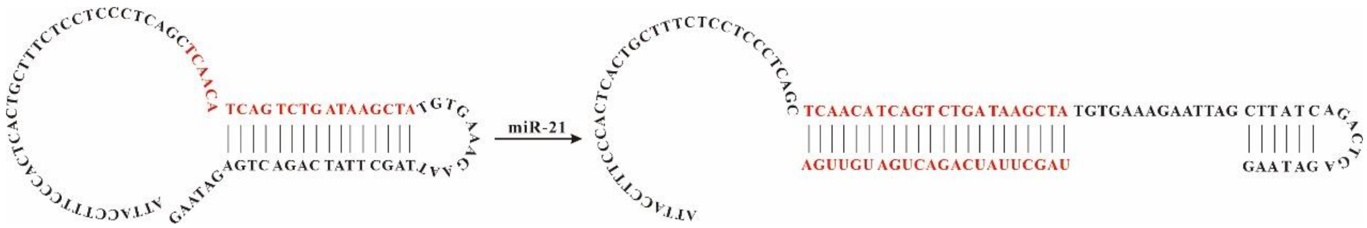

Figure S1. Schematic diagram of the conformational change occurring in the secondary structure of the SIAM reaction substrate HP-6nt before reaction and after binding to miR-21.

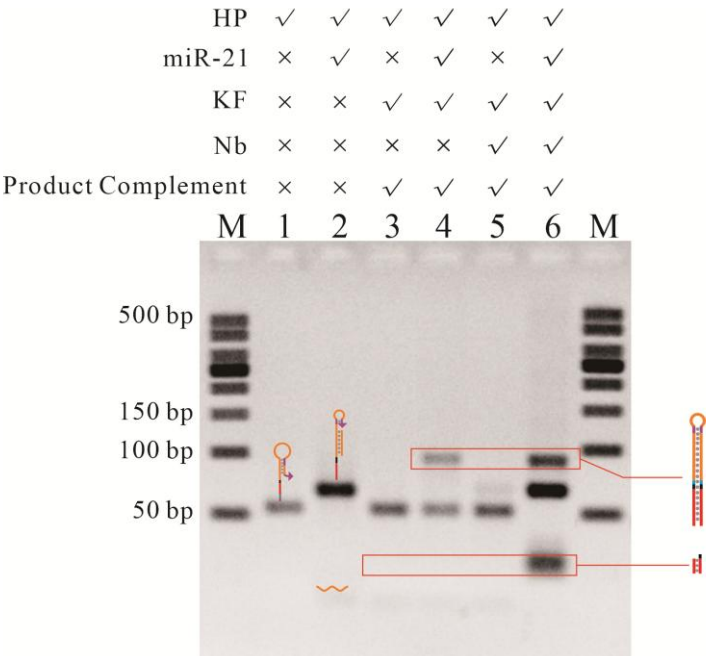

Figure S2. Agarose gel electrophoretic analysis of SIAM reaction. Lane 1: HP-6nt, Lane 2: HP-6nt+miR-21, Lane 3: HP-6nt+KF+Product Complement, Lane 4: HP-6nt+KF+Product Complement+miR-21, Lane 5: HP-6nt+KF+Nb+Product Complement, Lane 6: HP-6nt+KF+Nb+Product Complement+miR-21. The agarose gel electrophoresis parameters were as follows: 4% agarose gel, 100 V voltage, 70-min run time. The loading volume for the marker lane was 3 μL, while the loading volume for all other lanes was 6 μL. Except for lane 2, where the target concentration was 500 nM,

the target concentration in all other lanes was 5 nM. In addition, the concentration of HP was 500 nM, the reaction time of SIAM was 40 min, and the amounts of Nb and KF were 8 U and 2 U, respectively.

Table

Table S1. Sequence information of the oligonucleotides.

Synthetic Oligonucleotide	Sequence (5′→3′)
SP	SH-CGCACACGCACCGCATTTCCG-PO_4
HP-2nt	CCTTTCCCACTCACTGCTTTCTCCTC*CCTCAGC*TCAACATCAGTCTGATAAGC-TATGTGAAAGAATTAGCTTATCAGACTGAGATAAG
HP-6nt	ATTACCTTTCCCACTCACTGCTTTCTCCTC*CCTCAGC*TCAACATCAGTCTGATAAGC-TATGTGAAAGAATTAGCTTATCAGACTGAGATAAG
HP-10nt	TTCGATTACCTTTCCCACTCACTGCTTTCTCCTC*CCTCAGC*TCAACATCAGTCTGATAA-GCTATGTGAAAGAATTAGCTTATCAGACTGAGATAAG
HP-14nt	TTTTTTCGATTACCTTTCCCACTCACTGCTTTCTCCTC*CCTCAGC*TCAACATCAGTCTGA-TAAGCTATGTGAAAGAATTAGCTTATGAGATAAG
crRNA	UAAUUUCUACUAAGUGUAGAUCCACUCACUGCUUUCUCCUC
SIAM Product	TGAGGGAGGAGAAAGCAGTGAGTGGGAAAGGTAAT
miR-21	UAGCUUAUCAGACUGAUGUUGA
miR-199a	ACAGUAGUCUGCACAUUGGUUA
miR-141	UAACACUGUCUGGUAAAGAUGG
miR-155	UUAAUGCUAAUCGUGAUAGGGGU
Let-7a	UGAGGUAGUAGGUUGUAUAGUU
